# Minimally invasive plate osteosynthesis for humeral shaft nonunion: A report of two cases

**DOI:** 10.1016/j.amsu.2019.10.004

**Published:** 2019-10-11

**Authors:** Yoshihito Suda, Keisuke Oe, Tomoaki Fukui, Yutaka Mifune, Atsuyuki Inui, Teruya Kawamoto, Ryosuke Kuroda, Takahiro Niikura

**Affiliations:** Department of Orthopaedic Surgery, Kobe University Graduate School of Medicine, 7-5-1 Kusunoki-cho, Chuo-ku, Kobe, 650-0017, Japan

**Keywords:** Humeral shaft fracture, Nonunion, Minimally invasive plate osteosynthesis, Autogenous bone graft, Case report

## Abstract

**Introduction:**

We treated two cases of humeral shaft nonunion by minimally invasive plate osteosynthesis (MIPO) without autogenous bone grafting.

**Presntation of case:**

Case 1: An osteosynthesis with intramedullary nailing (IMN) was performed on a 17-year-old female for a humeral shaft fracture at another hospital; however, bony union was not obtained. We removed the nail and screws, then performed MIPO without autogenous bone grafting. At the final follow-up of 4 years after the surgery, she had obtained full range of motion.Case 2: Osteosynthesis with Rush pins had been performed in a 73-year-old female for a humeral shaft fracture at another hospital. Five months later, a revision surgery using IMN was performed at the same hospital; however, this led to nonunion. We removed the IMN and performed MIPO without autogenous bone grafting. At the final follow-up 2 years after surgery, she had obtained full range of motion.

**Discussion:**

The cause of nonunion is the lack of mechanical instability and/or biological activity. In these cases, from the findings of radiography and bone scintigraphy, mechanical instability was thought to be the primary cause; therefore, in order to enhance stability, we used a locking plate. Because we can see that these cases are biologically active, we decided not to use bone grafting. Both our cases successfully achieved bony union and excellent functional recovery using this method.

**Conclusion:**

We performed MIPO without exposure of the nonunion site and autogenous bone grafting in two cases of humeral shaft nonunion, and obtained successful clinical outcomes.

## Highlights

• To treat a nonunion, it is important to assess its causative factors.
• We treated two cases of humeral shaft nonunion due to the instability by MIPO without autogenous bone grafting.
• Both cases successfully achieved bony union and excellent functional recovery using this method.

## 1 Introduction

The frequency of nonunion following humeral shaft fracture ranges from 0.3% to 33% [[1], [2], [3]]. The standard treatment for humeral shaft nonunion is compression plate fixation and autogenous bone grafting [[3], [4], [5]]. Nevertheless, autogenous bone grafting might not necessarily be required in some cases. We treated two cases of humeral shaft nonunion by minimally invasive plate osteosynthesis (MIPO) without autogenous bone grafting.

## 2 Presentation of cases

According with the PROCESS criteria [1], we conducted a study of a consecutive series of 2 cases.

### 2.1 Case 1

**Fig. 1:**
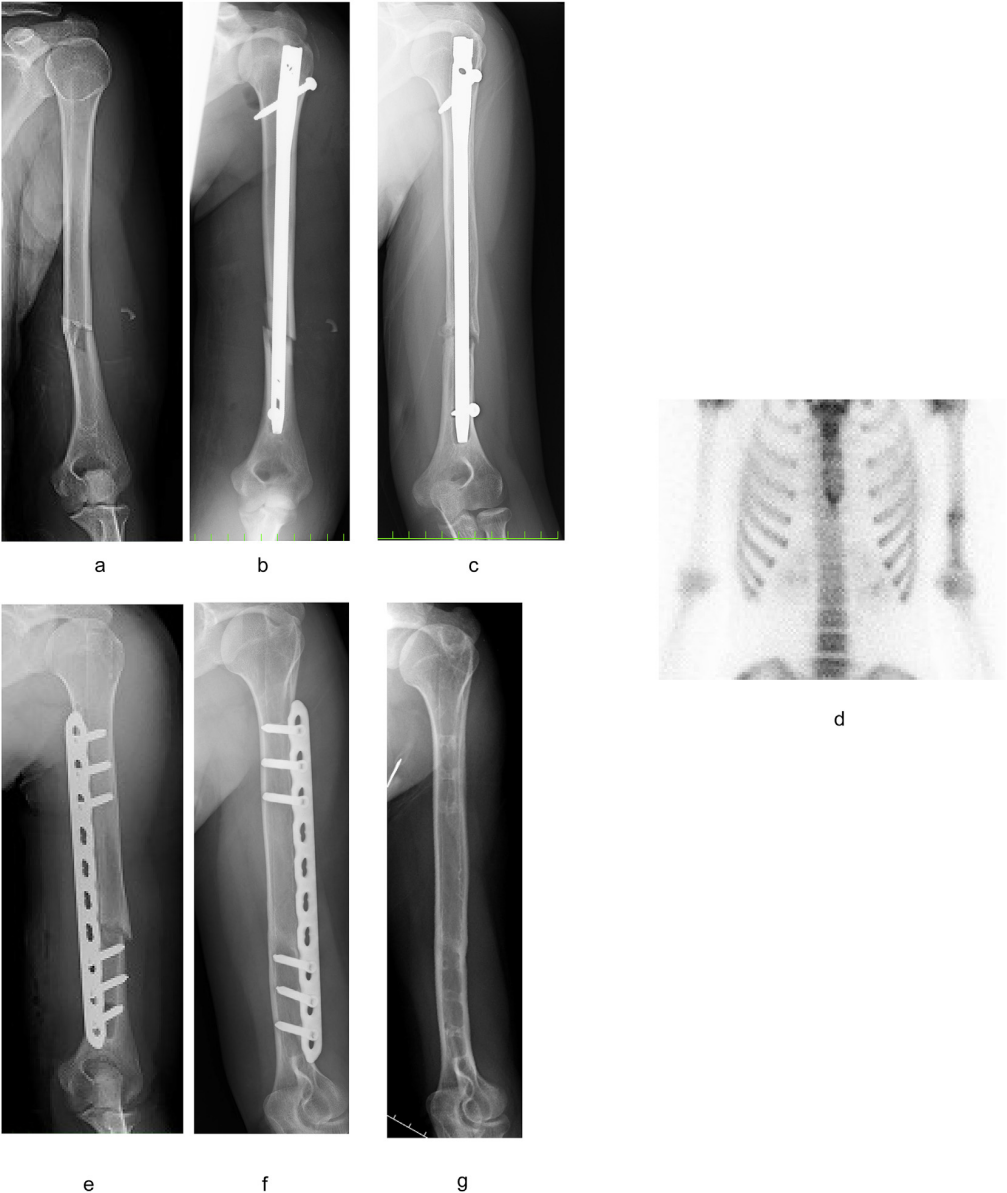
Views of Case 1. (a) AO classification: 12-B2 fracture. (b) Just after the 1st surgery with IMN. (c) 1 year after the 1st surgery. Little callus formation was observed around nonunion area. (d) Bone scintigraphy showed increased accumulation in the place of nonunion. (e) We performed the reoperation using the MIPO method without autogenous bone grafting. (f) One year after the surgery, bony union was obtained. (g) Final follow-up of 4 years after the surgery.

An osteosynthesis with intramedullary nailing (IMN) was performed on a 17-year-old female for a humeral shaft fracture at another hospital (Fig. 1a and b); however, bony union was not obtained. One year after the operation, she was referred to our hospital. A radiograph revealed little callus formation (Fig. 1c). Bone scintigraphy showed intense uptake at the nonunion site (Fig. 1d). We surmised that the primary causative factor of nonunion was lack of mechanical stability, especially in terms of rotational stability because of the small number of the interlocking screws. We removed the nail and screws, then performed MIPO without autogenous bone grafting (Fig. 1e). An anterior approach with two incisions was used. One distal approach reached the bone through the lateral side of the biceps. The musculocutaneous nerve appeared on the brachial muscle and the brachialis muscle was divided on the lateral side of the musculocutaneous nerve and medial side of the radial nerve. Another proximal approach passed between the deltoid and the biceps. Subsequently, a narrow locking compression plate (LCP) (DePuy Synthes, West Chester, USA) with 10 holes was slid onto the surface of the humerus from distal to proximal without exposure of the nonunion site. One year after surgery, bony union and good functional recovery was obtained (Fig. 1f), and we removed the implant. At the final follow-up of 4 years after the surgery (Fig. 1g), she had obtained full range of motion with no restrictions in activities of daily life.

### 2.2 Case 2

**Fig. 2:**
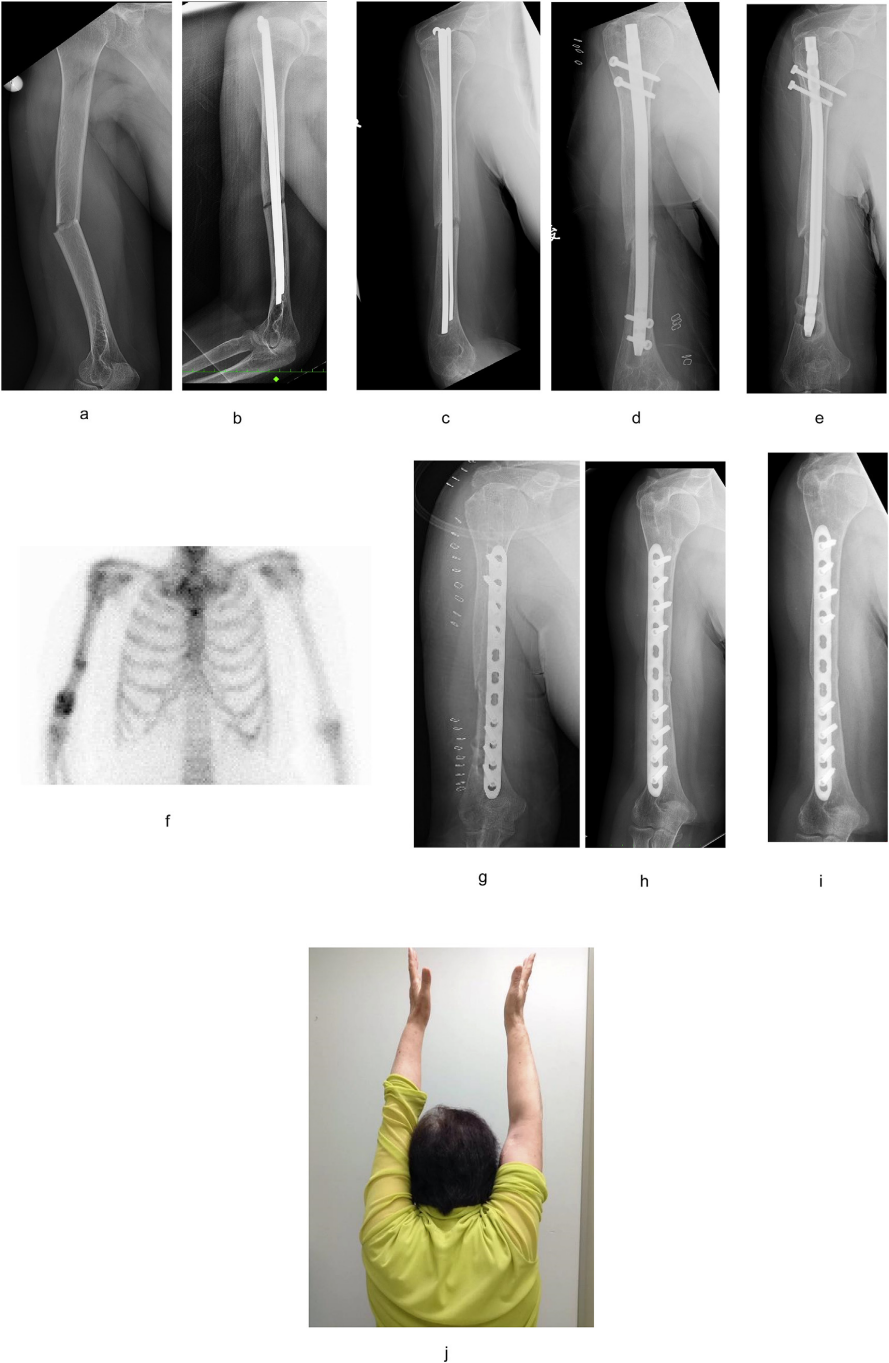
Views of Case 2. (2a) AO classification: 12A3 fracture. (2b) Just after the 1st surgery with RUSH pin. (2c) Five months after the 1st surgery. Diagnosed as nonunion. (2d) Exchange nailing was performed. (2e) One year after the 2nd surgery. Little callus formation was observed around nonunion area. (2f) Bone scintigraphy showed an increased accumulation in the place of nonunion. (2g) We performed the reoperation using the MIPO method without autogenous bone grafting. (2h) One year after the surgery, bony union was obtained. (2i) Final follow-up 2 years after the surgery. (2j) She obtained full range of motion with no restrictions in activities of daily life.

Osteosynthesis with Rush pins had been performed in a 73-year-old female for a humeral shaft fracture at another hospital (Fig. 2a and b). Five months later, a revision surgery using IMN was performed at the same hospital (Fig. 2c and d); however, this led to nonunion. Therefore, she was referred to our hospital one year after the second operation. A radiograph revealed little callus formation and loosening of the implant (Fig. 2e). Bone scintigraphy revealed intense uptake at the nonunion site (Fig. 2f). We surmised that the primary cause of this nonunion was lack of mechanical stability, similar to Case 1. We removed the IMN and performed MIPO without autogenous bone grafting using the same surgical technique as in Case 1 (Fig. 2g). One year after surgery, bony union and good functional recovery were achieved (Fig. 2h). We also observed restoration of the osteolysis around the distal screws. At the final follow-up 2 years after surgery (Fig. 2i), she had obtained full range of motion with no restrictions in activities of daily living (Fig. 2j).

## 3 Discussion

Nonunion after treatment of humeral shaft fractures occurs in 0.3%–33% of cases [[2], [3], [4]]. While it is reported that there are no significant differences among the three procedures (open reduction and plate fixation, IMN, and MIPO) in terms of frequency of nonunion, delayed union, or infection in the treatment of fresh humeral shaft fractures [7], autogenous bone grafting and compression plate fixation are the standard surgical procedures for humeral shaft nonunion [[4], [5], [6]]. The classification of Weber and Cech, which differentiates nonunions as vital or avital based on the underlying biological aspects, is widely utilized for determination of treatment of nonunions [8]. Recently, Giannoudis et al. listed four factors necessary for fracture healing: mechanical environment, stem cells, scaffolds, and growth factors. These four factors, summarized as the ‘Diamond concept’, are important factors in nonunion treatment [[9], [10]]. In other words, the cause of nonunion is considered from both biomechanical and biological perspectives. The diamond concept unites biomechanical and biological aspects and provides the pre-requisites for successful bone healing in nonunion [11].

In the two cases we presented, it appeared difficult to judge whether biological activity was preserved only on the basis of radiographs, because poor callus formation was observed. Niikura et al. mentioned that poor callus visualization (non-hypertrophic appearance) did not always indicate lack of biological activity; however, it is possible to evaluate the presence of biological activity by bone scintigraphy [12]. Another report indicated that uptake in bone scintigraphy reflects blood flow and new bone formation [13]. Uptake in bone scintigraphy was observed in both two cases, that is why we judged they had sufficient biological activity. Considering the mechanical stability in Case 1, screws were inserted only in the proximal and distal fragments. We judged the primary causative factor of this nonunion to be lack of rotational stability. In Case 2, at the time of the initial surgery, there was no anti-rotational stability. Therefore, mechanical instability due to insufficient rotational resistance was thought to be the cause of the initial nonunion. After the revision surgery using the IMN, two screws were inserted proximally and distally. Nevertheless, we realized that it was unstable on the basis of the loosening and osteolysis around the distal screws. IMN with two locking screws, both inserted into the proximal and distal fragments, was thought to be insufficient to fix this case of osteoporosis and the widened canal by the previously inserted Rush pins. We confirmed that infection was not the cause of the loosening and osteolysis by the intraoperative culture. Locking plate fixation seeks to maintain a certain elasticity to stimulate bone healing. In locking plate osteosynthesis, the quality of the reduction is less vital, provided that the local soft tissues are maintained intact. In that respect, the objectives are closer to the objectives of intramedullary nailing fixation. But locking plate fixation has better angular stability than intramedullary nails [14]. That is why we use locking plate for humeral shaft nonunion.

As previously mentioned, the cause of nonunion is the lack of mechanical instability and/or biological activity. It is thought that if one or both factors are involved, the fracture leads to nonunion. When treating nonunion, we consider the cause of each case and plan treatment accordingly. In these cases, from the findings of radiography and bone scintigraphy, mechanical instability was thought to be the primary cause; therefore, in order to enhance stability, particularly rotational stability, we used a locking plate. Because we can see that these cases are biologically active, we decided not to use bone grafting. Moreover, we wished to preserve the biological activity at the nonunion site including vascularity, and chose the MIPO technique without exposure of the nonunion site. The advantage of the MIPO is that it is less invasive for soft tissue and periosteum [15]. We found only one case series of treatment of humeral shaft nonunion by MIPO after an extensive literature search; in three cases of humeral shaft hypertrophic type nonunion, the authors performed osteosynthesis using the MIPO without autologous bone grafting, with good results [16]. Both our cases successfully achieved bony union and excellent functional recovery using this method.

## 4 Conclusion

To treat a nonunion, it is important to assess its causative factors. We performed MIPO without exposure of the nonunion site and autogenous bone grafting in two cases of humeral shaft nonunion, and obtained successful clinical outcomes.

## Ethical approval

This study was approved by the ethics committee of our hospital. (reference number: 180216).

## Sources of funding

None.

## Author contribution

All authors in this manuscript contributed to the interpretation of data, and drafting and writing of this manuscript. Yoshihito Suda is first and Takahiro Niikura is corresponding author of this paper. They and Keisuke Oe, Tomoaki Fukui conceived and designed the study and drafted the manuscript. Ryosuke Kuroda is a chief of orthopaedic surgery in our university hospital. All the authors read and approved the final manuscript.

## Research registration number

1. Name of the registry:Research Registry.

2. Unique Identifying number or registration ID:UIN researchregistry4861.

3. Hyperlink to the registration (must be publicly accessible): http://www.researchregistry.com.

## Guarantor

Takahiro Niikura, MD.

## Consent

Written informed consent was obtained from the patients for publication of this case report and accompanying images. A copy of the written consent is available for review by the Editor-in-Chief of this journal on request.

## Declaration of competing interest

None.
